# Block Copoly (Ester-Carbonate) Electrolytes for LiFePO_4_|Li Batteries with Stable Cycling Performance

**DOI:** 10.3390/ma17153855

**Published:** 2024-08-03

**Authors:** Yongjin Su, Bingyi Ma, Sheng Huang, Min Xiao, Shuanjin Wang, Dongmei Han, Yuezhong Meng

**Affiliations:** 1 School of Chemical Engineering and Technology, Sun Yat-Sen University, Guangzhou 510275, China; suyj25@mail2.sysu.edu.cn (Y.S.); maby@mail2.sysu.edu.cn (B.M.); 2 The Key Laboratory of Low-Carbon Chemistry & Energy Conservation of Guangdong Province/State Key Laboratory of Optoelectronic Materials and Technologies, School of Materials Science and Engineering, Sun Yat-Sen University, Guangzhou 510275, China; huangsh47@mail.sysu.edu.cn (S.H.); stsxm@mail.sysu.edu.cn (M.X.); wangshj@mail.sysu.edu.cn (S.W.); 3 Institute of Chemistry, Henan Academy of Sciences, Zhengzhou 450001, China; 4 College of Chemistry, Zhengzhou University, Zhengzhou 450001, China

**Keywords:** lithium metal batteries, block copolymers electrolytes, ionic conductivity

## Abstract

To address the challenges posed by the narrow oxidation decomposition potential window and the characteristic of low ionic conductivity at room temperature of solid polymer electrolytes (SPEs), carbon dioxide (CO_2_), epichlorohydrin (PO), caprolactone (CL), and phthalic anhydride (PA) were employed in synthesizing di-block copolymer PCL-b-PPC and PCL-b-PPCP. The carbonate and ester bonds in PPC and PCL provide high electrochemical stability, while the polyether segments in PPC contribute to the high ion conductivity. To further improve the ion conductivity, we added succinonitrile as a plasticizer to the copolymer and used the copolymer to assemble lithium metal batteries (LMBs) with LiFePO_4_ as the cathode. The LiFePO_4_/SPE/Li battery assembled with PCL-b-PPC electrolyte exhibited an initial discharge-specific capacity of 155.5 mAh·g^−1^ at 0.5 C and 60 °C. After 270 cycles, the discharge-specific capacity was 140.8 mAh·g^−1^, with a capacity retention of 90.5% and an average coulombic efficiency of 99%, exhibiting excellent electrochemical performance. The study establishes the design strategies of di-block polymer electrolytes and provides a new strategy for the application of LMBs.

## 1. Introduction

The application of lithium metal batteries (LMBs) is hampered by the hazardous thermal runaway due to the inevitable lithium dendrite growth in conventional liquid electrolytes [1,2]. The currently used liquid electrolytes struggle to achieve qualified safety performance [3,4]. Solid polymer electrolytes (SPEs) have been considered as a promising solution to address the aforementioned safety concerns due to their non-flammability and ability to inhibit the growth of lithium dendrites [5,6]. SPEs can also achieve high oxidation resistance and machinal strength through regulation of the polymer segments, thus enabling compatibility with cathode materials and lithium metal anodes [7,8,9]. Following the pioneering work of Armand et al., research on solid-state polymer batteries has made tremendous progress over the past decades [10].

However, polymer electrolytes still exhibit certain limitations. Typically, SPEs are composed of lithium salts and a polymer matrix capable of dissociating the lithium salts [11,12]. Poly(ethylene oxide) (PEO), due to its high dielectric constant (ε_r_ ≈ 5), relatively strong lithium ion solvation ability, and high chain segmental flexibility [13], has been one of the most extensively studied polymer electrolytes in recent years [14,15], but its limitations, such as a low lithium ion transference number (~0.2) [16], low ionic conductivity at room temperature (≈10^−6^ S·cm^−1^) [17], and a relatively narrow electrochemical stability window (vs. Li^+^/Li < 4.0 V), have limited further improvement of energy density in all-solid-state lithium batteries [18,19]. Several solutions have been proposed to address these challenges, including the use of polycarbonate-based polymers with good oxidation resistance, such as poly(ethylene carbonate) (PEC) [20,21], and poly(propylene carbonate) (PPC) [22,23,24]. Polycarbonate-based polymers can form complexes with lithium salts and contribute to the dissociation of Li^+^, thereby providing an ample number of free ions [25]. Moreover, PPC- and PEC-based polymer chains have good flexibility, which facilitates lithium ion transport and a higher electrochemical stability window (Li^+^/Li > 4.6 V) [26]. Alternatively, polyester-based polymers like poly(ε-caprolactone) (PCL) have also been explored as electrolytes [27]. Since C.P. Fonseca et al. first used PCL as a solid electrolyte in 2006, PCL has entered the field of solid-state batteries [28,29,30]. With its flexible chain structure, PCL exhibits robust Li^+^ solvation capability, a low glass-transition temperature (Tg = −60 °C), and a broad electrochemical stability window (5 V) [31]. Xue’s research group designed and synthesized a brush-like poly(ε-caprolactone) (PCL) electrolyte, which exhibited an ionic conductivity of 5.53 × 10^−5^ S·cm^−1^ at room temperature and a high lithium ion transference number (*t*_Li+_) of 0.82 at 60 °C [32]. However, PCL, as a semi-crystalline polyester, has a melting temperature range of 40 °C to 66 °C, and its ionic conductivity is limited, allowing it to act as an SPE only at high temperatures [33,34,35]. To modify the semi-crystallinity, various strategies have been employed, including the addition of plasticizers, the synthesis of graft polymers, the incorporation of nanoparticles into the polymer, and the synthesis of copolymers [12,36], where copolymers are formed by covalent bonds between two chemically distinct segments, thus combining the characteristics of the individual homopolymers to effectively improve the overall properties of SPEs. Xue et al. prepared block copolymers (BCPEs) by one-step synthesis of poly(ethylene glycol methyl ether acrylate) (PEGA) and ε-CL, and the polymer electrolytes exhibited an ionic conductivity of 1.09 × 10^−4^ S·cm^−1^ at room temperature with the *t*_Li+_ of 0.56. The block structure of BCPEs inhibits PCL crystallinity while maintaining a balanced polymer electrolyte for effective lithium salt dissociation and coordination, thereby enhancing both ionic conductivity and lithium ion transference [37]. 

In this study, AB-type di-block copolymers, namely PCL-b-PPCP and PCL-b-PPC, were formulated, where PCL represents the A-block, while PPC-P or PPC serves as the B-block. Flexible PPC has low glass-transition temperature and good interfacial compatibility, but it lacks mechanical properties. Phthalic anhydride groups can be introduced into the PPC chain to synthesize PPCP with excellent mechanical properties and thermal stability. PPC(P)-based chains have abundant carbonate groups, contributing to lithium salt dissociation and lithium ion conduction. PCL chains have robust Li^+^ solvation capability, low glass-transition temperature, and a broad electrochemical stability window. After the addition of succinonitrile (SN) as the plasticizer, the ionic conductivity of PCL-b-PPC can reach 1.99 × 10^−4^ S·cm^−1^ and exhibiting excellent electrochemical performance with a discharge capacity of 140.8 mAh·g^−1^ after 270 cycles at 0.5 C in the LiFePO_4_/SPE/Li full cell and maintaining a capacity retention of 90.5%. The copolymer has good mechanical properties and thermal properties and a low glass-transition temperature, with a lower cost than most of the polymers (such as PEO), indicating the potential for further application.

## 2. Materials and Methods

### 2.1. Materials

CO_2_ was purchased from GUANG QI (Guangzhou, China). Propylene oxide (PO, 99%), caprolactone (CL, 99%) and triethylborane (TEB, 1 mol/L in THF) were purchased from Energy (Shanghai, China). Succinonitrile (SN, 99%), ethyl methyl carbonate (EMC, 98%), ethylene carbonate (EC, 98%), *N*-methylpyrrolidone (NMP, 99.5%), and dimethyl carbonate (DMC, 98%) were obtained from Macklin (Shanghai, China). LiFePO_4_ (battery grade), Super P (battery grade), lithium bis((trifluoromethyl)sulfonyl) azalide (LiTFSI), and PP membrane (celguard 2500) were purchased from Canrd (Dongguan, China). Bis(triphenylphosphine)iminium chloride (PPNCL, 97%) was obtained from Alfa Aesar, Haverhill, MA, USA.

### 2.2. Preparation of PCL-b-PPCP and PCL-b-PPC

**Figure 1:**
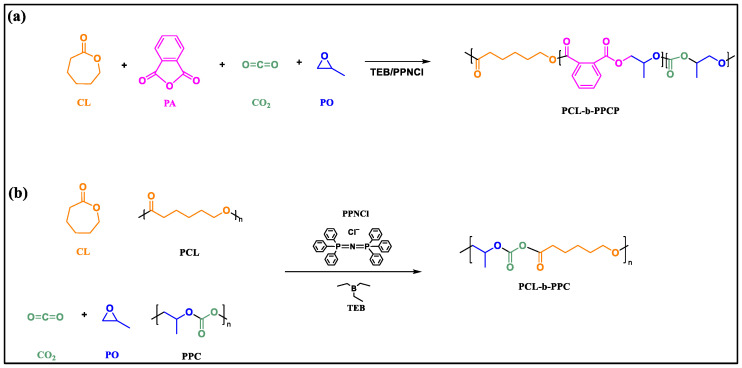
Synthetic schematic diagram of (**a**) PCL-b-PPCP and (**b**) PCL-b-PPC.

The synthesis of PCL-b-PPCP is shown in Figure 1a: A one-pot two-step method was used for the reaction. In a glove box, PO, CL, TEB, and PPNCl with a molar ratio of 500:100:0.1:1 were stirred at 80 °C for 48 h in the autoclave. After cooling to room temperature, the autoclave was brought back into the glove box, and PO, PA, CL, TEB, and PPNCl were added with a molar ratio of 500:50:100:1.4:1; the mixture was then sealed and taken out of the glove box, flushed with 1 MPa of CO_2_, and stirred at 60 °C for 4 h. After the reaction, the autoclave was cooled in an ice water bath and opened, and a sample was first taken for NMR test. Then, the reaction was quenched with 1 mol/L hydrochloric acid, the crude product was dissolved in dichloromethane and precipitated in ethanol, and the precipitated product was dried in a vacuum oven at 80 °C.

The synthesis of PCL-b-PPC is shown in Figure 1b: A one-pot two-step method was also used. In a glove box, PO, CL, TEB, and PPNCl were added to a 50 mL stainless-steel autoclave with a molar ratio of 1000:200:0.1:1; it was sealed and taken out of the glove box and stirred at 80 °C for 48 h. After cooling to room temperature, the autoclave was brought back into the glove box, and PO, CL, TEB, and PPNCl were added with a molar ratio of 1000:200:1.4:1; it sealed and taken out of the glove box, flushed with 1 MPa of CO_2_, and stirred at 50 °C for 4 h. The subsequent sampling and processing were the same as above.

Three polymers were selected as the base of the electrolyte membrane, among which PCL:PPC:PPO with a molar ratio of 37:52:11 was named PCL-b-PPC-1; PCL:PPCP:PPO = 38:50:12 was named PCL-b-PPCP-2; and PCL:PPCP:PPO = 28:61:11 was named PCL-b-PPCP-3. The molecular weights of these polymers were approximately 20,000. Three types of plasticizers are utilized: EMC, EC/EMC, and SN.

To prepare the polymer solution, 0.5 g of the above-mentioned polymer, 0.2 g of LiTFSI, and 0.3 g of the plasticizer were dissolved in 10 mL of dichloromethane. The solution was stirred until homogeneous. 

In a glove box, a small amount of the polymer solution was applied onto the positive electrode, and then, a celguard 2500 was placed on top. The polymer solution slowly filled the pores of the support membrane. The assembly was left at room temperature for 24 h to allow for solvent evaporation. Then, it was transferred to a heating pad and heated at 80 °C to remove the remaining solvent, resulting in a solid polymer electrolyte membrane.

### 2.3. Cathode Fabrication and Cell Assembly

LiFePO_4_, Super P, polyvinylidene fluoride (PVDF) in the weight ratio of 8:1:1 was dispersed in NMP and vigorously stirred for 6 h. The uniform slurry was cast onto the carbon-coated aluminum foils and dried under vacuum at 100 °C for 12 h to obtain the cathode foil. The CR2025-type cells were assembled for different test: LFP/solid electrolyte/Li, Li/solid electrolyte/Li (SE), steel sheet/solid electrolyte/steel sheet (SS), carbon paper electrode/solid electrolyte/Li (CL). Taking the LiFePO_4_/Li coin cells as an example. The LiFePO_4_/Li coin cells were assembled in the order of “positive electrode shell—LFP electrode—polymer solution—supporting membrane—lithium anode—steel sheet—shim—negative electrode shell”; specifically, the polymer solution was directly drop-coated onto the cathode, and the volatile solvent evaporated to form an electrolyte film on the cathode. Then, a support film was placed on top, allowing a small amount of electrolyte to infiltrate the electrode, creating a concentration gradient between the positive electrode and the electrolyte to enhance interface compatibility. The entire step was finished in an Ar-filled glove box (Mikrouna, Shanghai, China) with O_2_ and H_2_O contents below 0.1 ppm. After assembly, the cells were placed in a 60 °C blast oven for 24 h to enhance the interface stability between the electrode and electrolyte.

### 2.4. Electrochemical Measurements

The LiFePO_4_/SPE/Li cells were tested by Wuhan LAND battery system(LAND, Wuhan, China), and the coulombic efficiency was calculated as follows: (discharge-specific capacity/charge-specific capacity) × 100%. The ionic conductivity was measured by assembling SS for AC impedance spectroscopy measurements in the frequency range of 1 Hz–0.1 M Hz. The lithium transference number (*t_Li+_*) was measured by assembling SE through the method of AC impedance with DC polarization, and the ΔV = 10 mV. Linear sweep voltammetry was tested by assembling CL at a scan rate of 1 mV·S^−1^ from open-circuit voltage to 6 V. The ionic conductivity, lithium transference number, and linear sweep voltammetry were tested via an electrochemical workstation (Metrohm, Herisau, Switzerland). 

## 3. Results and Discussion

### 3.1. Analysis of the Structure and Thermal Properties of the Polymers 

**Figure 2:**
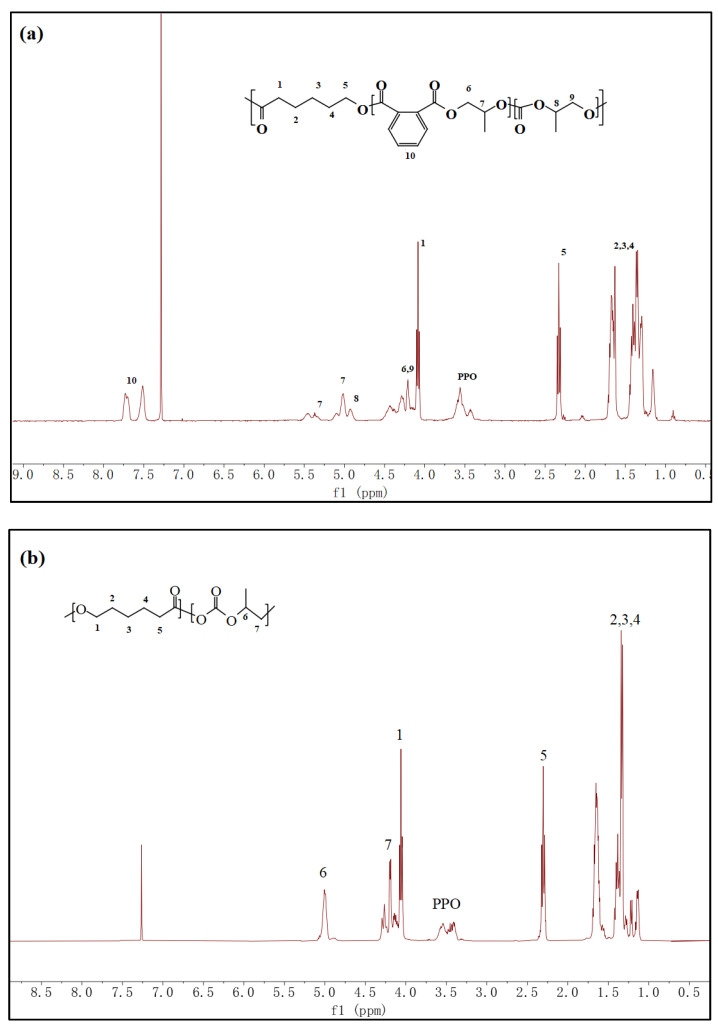
1H Spectra of (**a**) PCL-b-PPCP and (**b**) PCL-b-PPC.

As shown in Figure 2, the structures of purified PCL-b-PPCP and PCL-b-PPC copolymers were carefully analyzed using 1H NMR technique. In addition to the chemical shift from PPO, both PCL-b-PPCP and PCL-b-PPC could match all other chemical shifts.

**Figure 3:**
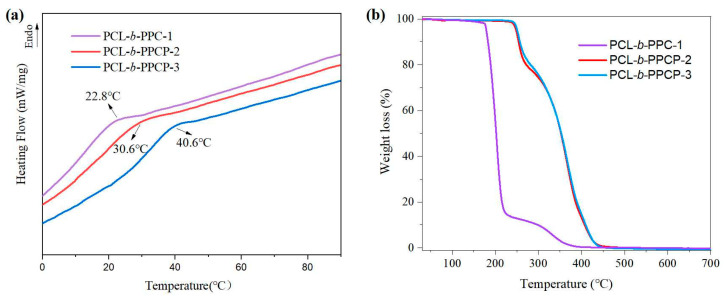
(**a**) DSC curves and (**b**) TGA curves of PCL-b-PPC-1, PCL-b-PPCP-2, and PCL-b-PPCP-3.

Compared to PCL-b-PPCP, due to the absence of phthalic anhydride, the glass-transition temperature (T_g_) of PCL-b-PPC-1 was lower (22.8 °C) (Figure 3a). This indicates that at the same temperature, PCL-b-PPC-1 has a larger free volume, faster chain segment movement rate, and more advantages in ion conduction. In comparison to PCL-b-PPCP-3, PCL-b-PPCP-2 owns more PCL segments, resulting in weakened interchain interactions and easier molecular chain movement, thereby reducing the glass-transition temperature.

Regarding the thermal stability of the polymers, the TGA curves in Figure 3b indicate that the PCL-b-PPC-1 began to decompose around 180 °C and completely decomposed at 300 °C, which could be attributed to the poor thermal stability of PPC. On the other hand, PCL-b-PPCP had a thermal decomposition temperature of 250 °C and complete decomposition at 450 °C, indicating that the introduction of PA enhanced the thermodynamic properties of the polymer. There is no significant difference in thermal stability between PCL-b-PPCP-2 and PCL-b-PPCP-3.

### 3.2. Electrochemical Performance Characterization of Electrolytes

EMC was selected as the plasticizer based on the principle of miscibility, while PP membrane was chosen as the support film. Then, 300 μL of the polymer solution was dispensed in the SS. This assembly was used to test the ion conductivity, and the test results are shown in Table 1. Without the addition of the plasticizer EMC, the ion conductivity of each electrolyte was very low (entries 1, 2, 5, 6, 9, and 10), measuring only 10^−7^ S·cm^−1^ at room temperature and 60 °C. When 30% wt.% EMC was added, the ion conductivity slightly increased but only by 10^−5^ S·cm^−1^ (entries 3 and 4). 

However, since the ionic conductivity of the aforementioned batteries at 60 °C was below 10^−4^ S·cm^−1^, which did not meet the threshold for battery operation, we replaced different plasticizers while keeping other conditions unchanged to investigate their effects on ionic conductivity. Table 2 shows the ionic conductivity of the electrolyte with EC/DMC (volume ratio of 1:1) as the plasticizer. Under the same conditions, EC/DMC exhibited higher ion conductivity than EMC. However, the ionic conductivity remained at 10^−5^ S·cm^−1^ at 60 °C, which was inadequate for normal battery operation. Furthermore, EC/DMC was replaced with succinonitrile (Table 3), and the ionic conductivity at 60 °C increased to 10^−4^ S·cm^−1^. This increase in ionic conductivity can be attributed to three factors: (1) the disordered molecular orientation of SN below the melting temperature, facilitating the transfer of Li^+^; (2) SN significantly reducing the crystallinity of the block copolymer, providing more amorphous regions that promote the transport of Li^+^ in the polymer matrix; and (3) the strong solvation and desolvation abilities of SN compared to ester-based plasticizers with strong solvation but weaker desolvation abilities, which may affect the movement of free Li^+^. Overall, compared to PCL-b-PPCP, the absence of benzoyl in PPC segments makes them more flexible, with a lower glass-transition temperature and stronger segment mobility, resulting in higher ionic conductivity of the PCL-b-PPCP-based electrolyte. Among them, PCL-b-PPC-1-SN, PCL-b-PPCP-2-SN, and PCL-b-PPCP-3-SN solid electrolytes were selected for further battery performance studies.

**Figure 4:**
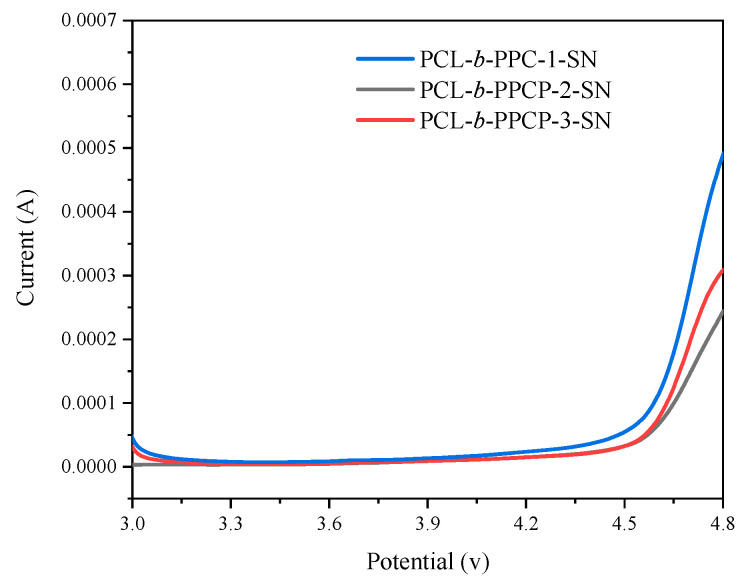
LSV profiles of three electrolyte membranes.

The electrochemical stability window of the electrolyte membrane was tested by linear sweep voltammetry (LSV) at 60 °C, as shown in Figure 4. The electrochemical decomposition of PCL-b-PPC-1-SN was 4.4 V, slightly lower than PCL-b-PPCP-2-SN and PCL-b-PPCP-3-SN (4.5 V), which paved the way to improve the adaptability between solid electrolytes and high-voltage cathode materials and has broad application prospects in high-voltage solid-state lithium batteries.

**Figure 5:**
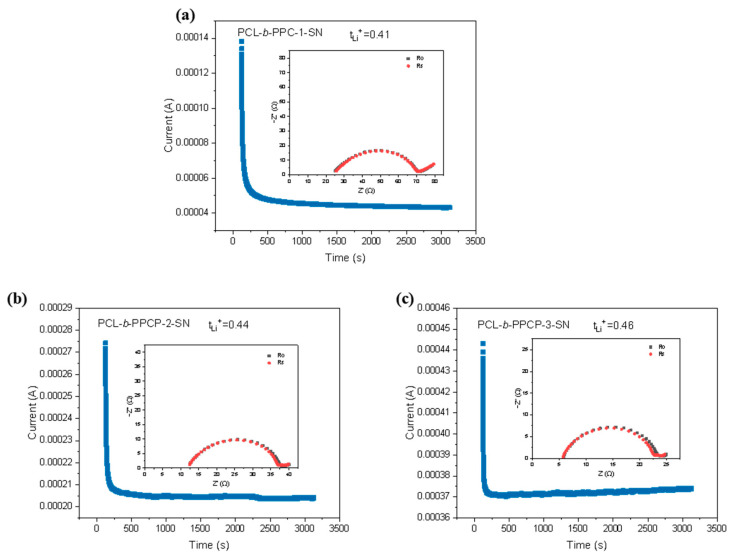
DC polarization curves of (**a**) PCL-b-PPC-1-SN, (**b**) PCL-b-PPCP-2-SN, and (**c**) PCL-b-PPCP-3-SN electrolyte membrane at 60 °C (Inset: The EIS spectra before and after polarization).

The lithium-ion transference number (*t*_Li+_) was determined by a DC polarization combined with impedance spectroscopy by SE cells. The tests results are shown in Figure 5. The initial current of PCL-b-PPC-1-SN was 0.138 mA, which reached a steady state after 1000 s with a stable current of 0.044 mA. The interface impedance before polarization was 21.5 Ω, and 20.4 Ω after polarization, and the *t*_Li+_ was calculated to be 0.41 according to Bruce–Vincent equation. The LTN for PCL-b-PPCP-2-SN and PCL-b-PPCP-3-SN (Figure 5b,c) was 0.44 and 0.46, respectively. The high *t*_Li+_ is mainly attributed to the addition of SN, which promotes the generation of large amorphous regions and enables the transference of Li^+^. Furthermore, the electronegative N atoms in SN restrict the accumulation of anions, resulting in reduced polarization and increased *t*_Li+_ in PCL-b-PPCP and PCL-b-PPC electrolytes.

**Figure 6:**
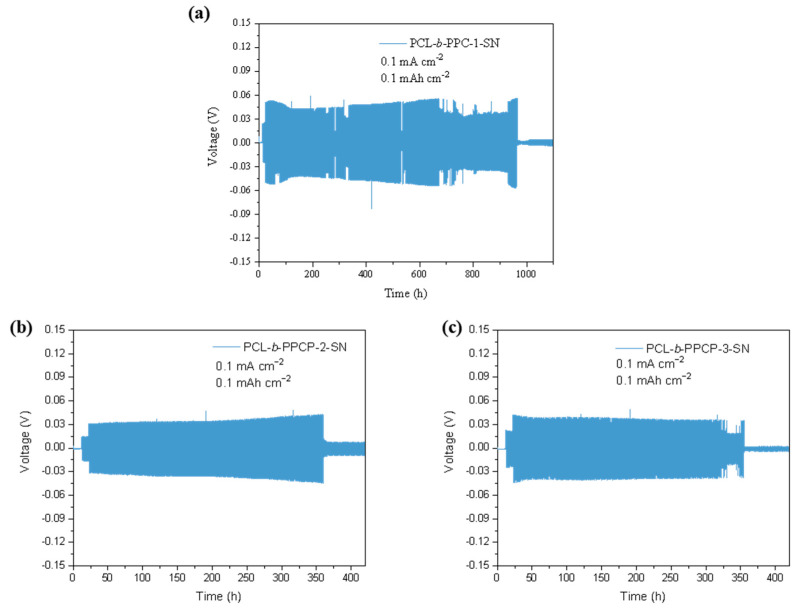
(**a**) Li/PCL-b-PPC-1-SN/Li, (**b**) Li/PCL-b-PPCP-2-SN/Li, and (**c**) PCL-b-PPCP-3-SN/Li symmetric battery exhibits deposition stripping performance at 0.1 mA·cm^−2^.

To investigate the cycling stability and inhibition ability of lithium dendrites, the symmetric cells with PCL-b-PPC-1-SN, PCL-b-PPCP-2-SN, and PCL-b-PPCP-3-SN electrolyte were assembled and tested at 60 °C and 0.1 mA·cm^−2^. As shown in Figure 6, the Li/PCL-b-PPC-1-SN/Li symmetric cell exhibited stable cycling for over 900 h with an overpotential of approximately 0.06 V. The Li/PCL-b-PPCP-2-SN/Li symmetric cell showed stable cycling for 350 h with a stable overpotential of 0.04 V. The Li/PCL-b-PPCP-3-SN/Li symmetric cell exhibited stable cycling for 320 h with an overpotential of 0.04 V. No apparent short-circuit or micro-short-circuit events were observed during the cycling process. Compared to PCL-b-PPCP-2-SN, the PCL-b-PPC-1-SN electrolyte was softer, exhibited better interface compatibility with lithium, and had higher ionic conductivity, which facilitated uniform deposition and stripping of lithium and suppressed the formation of lithium dendrites.

**Figure 7:**
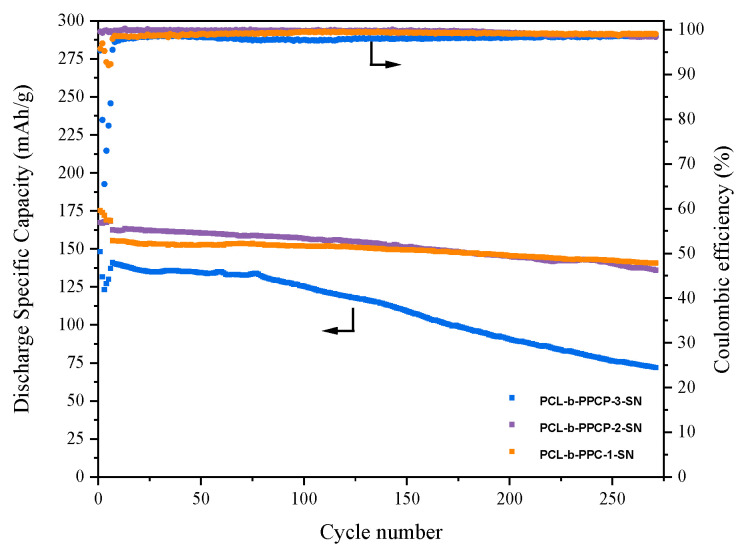
Cycle performance (Left vertical axis) and coulombic efficiency (right vertical axis) of LFP/PCL-b-PPC-1-SN/Li, LFP/PCL-b-PPCP-2-SN/Li, and LFP/PCL-b-PPCP-3-SN/Li batteries at 0.5 C and 60 °C.

To investigate the feasibility of the polymer electrolytes for practical application in the full cells consisting of LFP cathode, different polymer electrolytes and Li-metal anodes were assembled. The cycling performances of the cells were conducted at a C-rate of 0.5 C and a temperature of 60 °C, with the first six cycles serving as an activation process and with a charge–discharge at 0.1 C. As shown in Figure 7, the LFP/PCL-b-PPC-1-SN/Li exhibited a high initial discharge capacity of 155.5 mAh·g^−1^ at 0.5 C and can maintain a capacity of 140.8 mAh·g^−1^ after 270 cycles, resulting in a capacity retention of 90.5% and an average coulombic efficiency of 99%. LFP/PCL-b-PPCP-2-SN/Li exhibited an initial discharge-specific capacity of 168.2 mAh·g^−1^ at 0.5 C. After 270 cycles, the discharge capacity decreased to 136.5 mAh·g^−1^, resulting in a capacity retention of 81.2%, with a coulombic efficiency above 98%. And the LFP/PCL-b-PPCP-3-SN/Li showed 137.1 mAh·g^−1^ initial discharge capacity, 121.7 mAh·g^−1^ after 110 cycles, and 73.3 mAh·g^−1^ after 270 cycles. The PCL-b-PPCP electrolyte showed lower capacity retention than PCL-b-PPC owing to the poor stability at electrode/electrolyte interfaces, which resulted in larger polarization and the severe growth of lithium dendrites. These results indicated that the presence of rigid benzene in the chain segments is not conducive to the oscillation of polymer chain segments, thereby affecting the electrochemical performance of electrolytes. Additionally, the long retention of coulombic efficiency at a high value in the LFP/PCL-b-PPC-1-SN/Li cell was attributed to the much better electrochemical stability of PCL-b-PPC-1-SN compared to PCL-b-PPCP-2-SN and PCL-b-PPCP-3-SN. Accordingly, the PCL-b-PPC-1-SN effectively suppressed the side reaction with the electrodes, contributing to the consistently maintained coulombic efficiency of 99% in the LFP/PCL-b-PPC-1-SN/Li cell throughout the long cycles.

**Figure 8:**
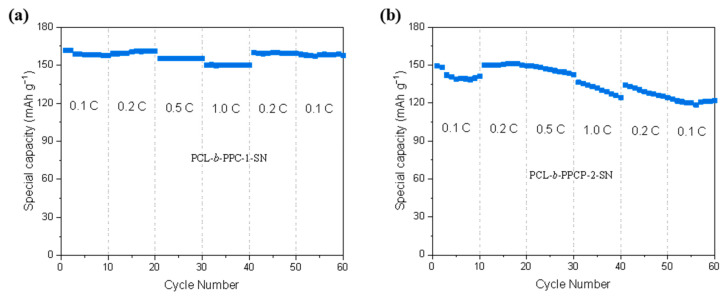
Rate performance of (**a**) LFP/PCL-b-PPC-1-SN/Li and (**b**) LFP/PCL-b-PPCP-2-SN/Li at 60 °C.

Rate capability is a crucial factor in battery commercialization, as people demand fast charging. Figure 8 showed the rate performance of LFP/PCL-b-PPC-1-SN/Li and LFP/PCL-b-PPCP-2-SN/Li with a range of C-rates (0.1−1 C) at 60 °C. The initial discharge-specific capacities of LFP/PCL-b-PPC-1-SN/Li at different rates are 161.8 mAh·g^−1^ (0.1 C), 159.1 mAh·g^−1^ (0.2 C), 155.4 mAh·g^−1^ (0.5 C), 150.1 mAh·g^−1^ (1.0 C), and 159.9 mAh·g^−1^ (0.2 C), and when returned to 0.1 C, the capacity was restored to a value (158.5 mAh·g^−1^) similar to the initial discharge capacity of 161.8 mAh·g^−1^. The outstanding capacity restoration indicates the excellent electrochemical stability of the PCL-b-PPC-1-SN. On the other hand, the initial discharge-specific capacities of LFP/PCL-b-PPCP-2-SN/Li at different charge/discharge rates are 149.5 mAh·g^−1^ (0.1 C), 149.8 mAh·g^−1^ (0.2 C), 149.3 mAh g^−1^ (0.5 C), 136.3 mAh·g^−1^ (1.0 C), mAh·g^−1^ (0.2 C), and 122.9 mAh·g^−1^ (0.1 C). The capacity rapidly decreases at the same rates, possibly due to high interface impedance and dendritic lithium. When the current returns to 0.1 C, the specific capacity is slightly lower compared to the previous cycles, indicating poor rate performance. In conclusion, PCL-b-PPC-1-SN exhibited better rate performance than the PCL-b-PPCP-2-SN.

## 4. Conclusions

To address the critical issues of low ionic conductivity and narrow oxidation decomposition potential window for polymer electrolytes, three block copolymers, PCL-b-PPC-1, PCL-b-PPCP-2, and PCL-b-PPCP-3 were used in a one-pot, two-step method in this work. The carbonate and ester bonds in PPC and PCL provide high electrochemical stability, and the polyether segments in PPC contribute to the high ion conductivity, which can help to overcome the aforementioned challenges. These copolymers were matched with different plasticizers to in situ generate uniform electrolyte membranes on the electrodes, resulting in enhanced lithium ion conductivity. Compared to PCL-b-PPCP-2 and PCL-b-PPCP-3, PCL-b-PPC-1 has a lower *T*_g_ and more flexible chain segments, which are beneficial for lithium ion conduction. And attributed to the high dielectric constant, SN showed a better improvement in ion conductivity compared to other plasticizers. After addition of SN, the ion conductivities of PCL-b-PPC-1-SN, PCL-b-PPCP-2-SN, and PCL-b-PPCP-3-SN at 60 °C were 1.99 × 10^−4^ S·cm^−1^, 1.25 × 10^−4^ S·cm^−1^, and 1.14 × 10^−4^ S·cm^−1^; the *t*_Li+_ were 0.41, 0.44, and 0.46, respectively. Therefore, the Li/PCL-b-PPC-1-SN/Li symmetric cell exhibited stable cycling for over 900 h at a current density of 0.1 mA·cm^−2^, with an overpotential of approximately 0.06 V, demonstrating excellent interface stability with lithium metal. Furthermore, the full cell assembled by LFP/PCL-b-PPC-1-SN/Li showed an initial discharge-specific capacity of 155.5 mAh·g^−1^ at 0.5 C, and after 270 cycles, the discharge-specific capacity was 140.8 mAh·g^−1^, indicating good cycle stability. Our work provides a good optimization approach for solid-state electrolyte in lithium metal batteries.

## Figures and Tables

**Table 1:** The ion conductivity of the polymer electrolytes with 30 wt.% EMC.

Entry	Sample	Temperature/°C	Thickness/μm	R/Ω	σ/S cm^−1^
1 ^a^	PCL-*b*-PPC-1	25	45	7600	3.10 × 10^−7^
2 ^a^	PCL-*b*-PPC-1	60	45	645	3.65 × 10^−6^
3 ^b^	PCL-*b*-PPC-1-EMC	25	36	165	1.14 × 10^−5^
4 ^b^	PCL-*b*-PPC-1-EMC	60	36	55	3.45 × 10^−5^
5 ^a^	PCL-*b*-PPCP-2	25	51	66,000	4.04 × 10^−8^
6 ^a^	PCL-*b*-PPCP-2	60	51	6700	3.98 × 10^−7^
7 ^b^	PCL-*b*-PPCP-2-EMC	25	35	810	2.26 × 10^−6^
8 ^b^	PCL-*b*-PPCP-2-EMC	60	35	134	1.37 × 10^−5^
9 ^a^	PCL-*b*-PPCP-3	25	50	49,000	5.34 × 10^−8^
10 ^a^	PCL-*b*-PPCP-3	60	50	1640	1.60 × 10^−6^
11 ^b^	PCL-*b*-PPCP-3-EMC	25	39	1260	1.62 × 10^−6^
12 ^b^	PCL-*b*-PPCP-3-EMC	60	39	194	1.05 × 10^−5^

^a^: Polymer electrolytes without plasticizer. ^b^: The polymer electrolyte is composed of 50 wt.% polymer, 30 wt.% EMC, and 20 wt.% LiTFSI.

**Table 2:** Ionic conductivity of polymer electrolyte with 30 wt.% EC/DMC.

Entry	Sample	Temperature/°C	Thickness/μm	R/Ω	σ/S cm^−1^
1	PCL-*b*-PPC-1-EC/DMC	25	34	98	1.82 × 10^−5^
2	PCL-*b*-PPC-1-EC/DMC	60	34	45	3.96 × 10^−5^
3	PCL-*b*-PPCP-2-EC/DMC	25	46	167	1.44 × 10^−5^
4	PCL-*b*-PPCP-2-EC/DMC	60	46	79	3.05 × 10^−5^
5	PCL-*b*-PPCP-3-EC/DMC	25	41	688	3.12 × 10^−6^
6	PCL-*b*-PPCP-3-EC/DMC	60	41	128	1.68 × 10^−5^

The polymer electrolyte is composed of 50 wt.% polymer, 30 wt.% EC/DMC, and 20 wt.% LiTFSI.

**Table 3:** Ionic conductivity of polymer electrolyte with 30 wt.% SN.

Entry	Sample	Temperature/°C	Thickness/μm	R/Ω	σ/S cm^−1^
1	PCL-*b*-PPC-1-SN	25	40	40	5.24 × 10^−5^
2	PCL-*b*-PPC-1-SN	60	40	11	1.99 × 10^−4^
3	PCL-*b*-PPCP-2-SN	25	31	47	3.42 × 10^−5^
4	PCL-*b*-PPCP-2-SN	60	31	13	1.25 × 10^−4^
5	PCL-*b*-PPCP-3-SN	25	32	73	2.29 × 10^−5^
6	PCL-*b*-PPCP-3-SN	60	32	15	1.14 × 10^−4^

The polymer electrolyte is composed of 50 wt.% polymer, 30 wt.% SN, and 20 wt.% LiTFSI.

## Data Availability

The original contributions presented in the study are included in the article; further inquiries can be directed to the corresponding authors.
